# Frailty and cerebrovascular disease: Concepts and clinical implications for stroke medicine

**DOI:** 10.1177/17474930211034331

**Published:** 2021-08-04

**Authors:** Nicholas R Evans, Oliver M Todd, Jatinder S Minhas, Patricia Fearon, George W Harston, Jonathan Mant, Gillian Mead, Jonathan Hewitt, Terence J Quinn, Elizabeth A Warburton

**Affiliations:** 1 Department of Medicine, University of Cambridge, Cambridge, UK; 2 Academic Unit for Ageing and Stroke Research, University of Leeds, Leeds, UK; 3 NIHR Leicester Biomedical Research Centre, University of Leicester, Leicester, UK; 4 Department of Stroke Medicine, Royal Victoria Hospital, Belfast, UK; 5 Acute Stroke Programme, Oxford University Hospitals NHS Foundation Trust, Oxford, UK; 6 Department of Public Health & Primary Care, University of Cambridge, Cambridge, UK; 7 Centre for Clinical Brain Sciences, University of Edinburgh, Edinburgh, UK; 8 Division of Population Medicine, Cardiff University, Cardiff, UK; 9 Institute of Cardiovascular and Medical Sciences, University of Glasgow, Glasgow, UK; 10 Department of Clinical Neurosciences, University of Cambridge, Cambridge, UK

**Keywords:** Frailty, stroke, inflammageing, rehabilitation

## Abstract

Frailty is a distinctive health state in which the ability of older people to cope with acute stressors is compromised by an increased vulnerability brought by age-associated declines in physiological reserve and function across multiple organ systems. Although closely associated with age, multimorbidity, and disability, frailty is a discrete syndrome that is associated with poorer outcomes across a range of medical conditions. However, its role in cerebrovascular disease and stroke has received limited attention. The estimated rise in the prevalence of frailty associated with changing demographics over the coming decades makes it an important issue for stroke practitioners, cerebrovascular research, clinical service provision, and stroke survivors alike. This review will consider the concept and models of frailty, how frailty is common in cerebrovascular disease, the impact of frailty on stroke risk factors, acute treatments, and rehabilitation, and considerations for future applications in both cerebrovascular clinical and research settings.

## Introduction

**Figure 1.**
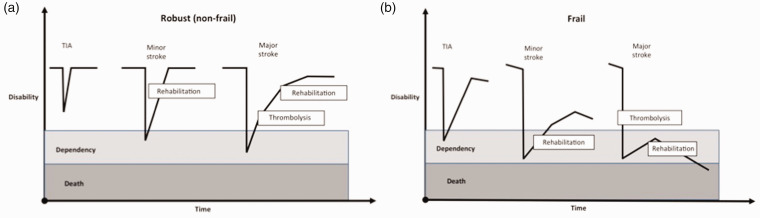
Differing trajectories in disability following stroke events in non-frail (a) and frail (b) individuals.

Frailty—the state of vulnerability characterized by the cumulative multisystem decline of physiological reserves to maintain homeostasis following a stressor event^1^—is associated with increased morbidity and mortality across a range of medical conditions,^2^ though only recently has attention been paid to its role in cerebrovascular disease. Stroke represents an archetypal stressor event, and frailty may affect stroke risk factors, disease trajectory, and outcomes (Figure 1).

**Figure 2.**
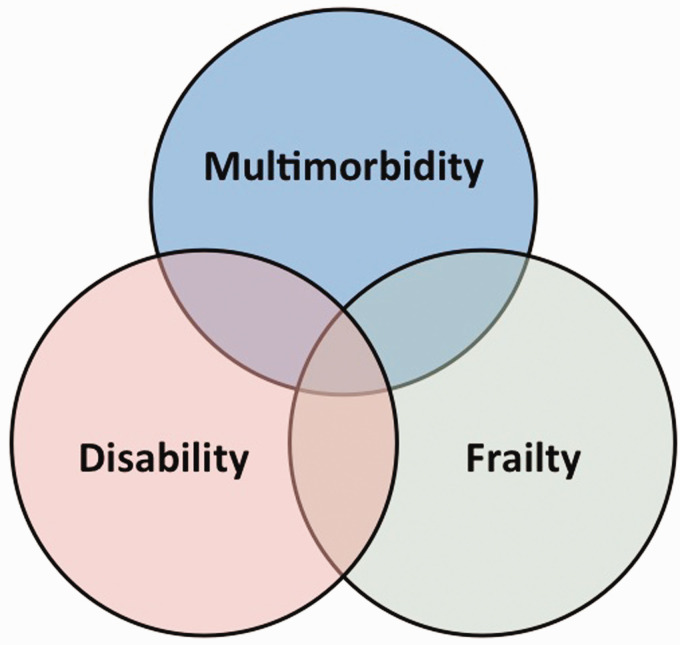
Schema illustrating the relationships between frailty, disability, and multimorbidity.

Frailty is a distinct clinical syndrome discrete from—but closely related to—age, multimorbidity, and disability (Figure 2). Although these conditions frequently co-exist, an individual may be frail in the absence of significant co-morbidity and disability, and without being elderly. This distinction is important, as it may be possible to attenuate or reverse frailty trajectories in order to reduce its burden on health outcomes.^3^

The prevalence of frailty rises markedly with age.^4^ However, as people are living longer, and living for an extended proportion of that time with greater disability and comorbidity, there is a wide variation in the health of older people. Chronological age is insufficient to capture this variation in the ageing process. Despite advocacy of the “Compression of Morbidity” paradigm—where postponement of chronic disease outweighs any increase in life-expectancy, thereby reducing time in later life with chronic disability^5^—some western countries have experienced worsening health across multiple age ranges.^6^ Shifting demographic trends with rising numbers of older, multimorbid, frailer individuals necessitate a move away from consideration of single organ disease-specific processes to a more nuanced frailty-based consideration of how the multisystem decline in physiological reserves and consequent vulnerability modifies the natural history of stroke.

This review will consider the models of frailty and how it is evaluated, prior to considering the effect of frailty along the natural history of stroke (including effects on cardiovascular risk factors preceding stroke, its role during acute stroke presentation and treatment, and impact after stroke on rehabilitation and secondary prevention). Finally, we will consider future directions and applications for frailty in both clinical care and research.

## Concepts of frailty

Two predominant approaches to evaluating frailty have developed based around measuring deficits versus assessing a frailty phenotype.

### Cumulative deficit model

This operationalized model of frailty considers that “the more things individuals have wrong with them, the higher the likelihood that they will be frail.”^7^ This model is predicated upon recognition that physiological changes (“deficits”) may not necessarily achieve disease status, yet their accumulation is associated with higher levels of frailty and adverse outcomes. The Cumulative Deficit Model quantifies frailty through a frailty index consisting of a number of equally weighted deficits across different domains (including cognition, function, mobility, and continence), where the number of deficits present in the individual is divided by the total number of possible scoring deficits to give a ratio between zero to one which reflects the spectrum of frailty (Table 1). Frailty, as defined by this deficit accumulation, is associated with increased mortality and rates of institutionalization.^8^

**Table 1.** Exemplar of a frailty index used in individuals presenting with stroke.^14^

Frailty index		
Depression Anxiety Polypharmacy Previous cerebrovascular disease Atrial fibrillation Diabetes Hypertension Previous myocardial infarction Heart failure Vascular disease Hyperlipidaemia	Haemoglobin (low) Care-home resident Carers Hearing aid Sensory impairment (e.g. blind/deaf) Continence bladder Continence bowel Falls Fracture Chronic Obstructive Pulmonary Disease Cancer	Liver disease Peptic ulcer Arthritis Impaired external ADL Impaired ADL Mobility aid Assistance walking Calcium Albumin (low) High glucose Renal failure

Note: This approach considers equally weighted deficits across different domains (including function, mobility, continence, co-morbidities, and biochemical values). To be robust, a frailty index should have approximately 30–40 potential deficits spanning multiple domains.

### The frailty phenotype model

In contrast to the Cumulative Deficit Model, the Fried Phenotype Model recognizes five main phenotypical characteristics of frailty:

1. Weight loss
2. Self-reported exhaustion
3. Low levels of activity
4. Slow gait speed
5. Weak grip strength.

When comparing those with no criteria (non-frail), one or two criteria (intermediate frailty), and three or more criteria (frail) in an unselected population, there is a clear increase in mortality with increasing frailty, as well as associations with falls, worsening mobility, functional disability, and hospitalization.^9^

### Measuring frailty

Recognition of the importance of frailty has resulted in policymakers advocating frailty screening in unscheduled admissions. The measures used may reflect:

1. Different models of frailty: frailty indices (Cumulative Deficit Model), or measures including grip strength and walking speed (Frailty Phenotype Model).
2. Different clinical contexts:

- Secondary care: The Hospital Frailty Risk Score (HFRS) considers 109 routinely collected ICD-10 diagnoses to produce a score associated with length of hospitalization and in-patient mortality.^10^
- Community: The electronic frailty index (eFI) using 36 deficits in primary health datasets measures frailty at a population level, and demonstrates associations with hospitalization, nursing home admission, and all-cause one-year mortality.^11^

1. Different data settings:

- Bedside assessment using the Clinical Frailty Scale (CFS), which correlates strongly with the frailty index, evaluates how an individual aged over 65 years was, two weeks prior to admission (and importantly not as they appear at time of admission).^12^
- Routinely collected health data, e.g. eFI, HFRS.
- Research study data, e.g. grip strength, gait speed.

Although premorbid modified Rankin Scale (mRS) is frequently used to determine eligibility for participation in stroke clinical trials, it is important to recognize that pre-stroke mRS (a measurement of disability) is not a substitute for frailty assessment. Pre-stroke mRS demonstrates reasonable agreement with a frailty index, though only one-third of individuals had evidence of frailty yet over half were classed as dependent on pre-stroke mRS, and there was a cohort with frailty but low disability on the pre-stroke mRS.^13^ Other studies have reported moderate agreement between pre-stroke mRS and a frailty index, but only slight agreement between pre-stroke mRS and phenotypical frailty measurements.^14^ However, other studies have reported no statistically significant correlation between CFS and mRS.^15^ Future work needs to consider the best method for evaluating frailty in the stroke setting.

Such considerations highlight a challenge for the operationalized use of frailty measurements: there remains debate over whether frailty should be considered according to individual domains (physical, cognitive, brain appearances) or the total burden of frailty for the individual. The relative weighting of these different domains vary within different frailty scales, and consequently may make direct comparisons between studies challenging. This review will consider the total burden of frailty on the individual, but will explore the associations described with different frailty domains. Arguably, the abundance of neuroimaging in Stroke may facilitate the operationalized radiological evaluation of “brain frailty,” but for the clinician seeing the patient it is often the totality of frailty that is important. The relative strengths and weaknesses of a total versus sub-type evaluation of frailty, and whether these vary according to the aspect of stroke care represent important avenues of future research into the biological mechanisms underlying the impact of frailty in stroke etiology and outcomes.

## Frailty and vascular risk factors

Frailty is associated with increasing 10-year Framingham Risk Scores, with Scores particularly pronounced in the presence of weight loss, weakness, and slowness components of the frailty phenotype.^16^ Frailty frequently co-exists with conventional cardiovascular risk factors, where it demonstrates disease-modifying and treatment-modifying effects. In hypertension, achieving a systolic blood pressure below 140 mmHg was associated with a 14% reduction in all-cause mortality in non-frail individuals, yet no difference in all-cause mortality was seen in frailer individuals.^17^ In individuals with diabetes, frailty is associated with increased mortality, hospital admission, disability, and cognitive impairment.^18^ Atherosclerotic burden is also associated with frailty,^19^ potentially through sub-clinical effects on end-organs contributing to decreased function and physiological reserve. As discussed in subsequent sections, such end-organ effects on the brain may contribute to findings of “brain frailty” and negatively impact cognitive reserve.

Frailty is common in individuals with atrial fibrillation, with approximately two-thirds of individuals being pre-frail or frail, and independently associated with higher rates of hospitalization, all-cause mortality, bleeding, and stroke.^20^ Frailty is associated with lower odds of being prescribed anticoagulation at the time of hospitalization, but higher odds of being prescribed anticoagulation in community settings.^21^ Additionally, frailty is a major factor influencing discontinuation of therapy for those already taking anticoagulants.^22^ Such findings illustrate the perpetual dilemma for prescribing anticoagulation in co-existent atrial fibrillation, frailty, and risk of falls, particularly given the rising rate-adjusted fall death rate as more individuals are surviving with stroke disability.^23^

## Frailty and the risk of stroke

Frailty in stroke is common. A recent meta-analysis of 18 studies with 48,009 participants reported the prevalence of pre-frailty and frailty in individuals with stroke as 49% and 22%, respectively.^24^ Frail individuals with stroke are typically older and more likely to be female.^25^

**Figure 3.**
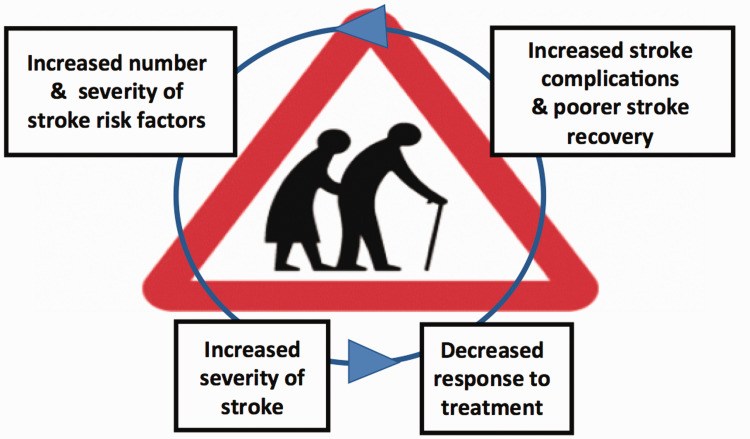
Factors influencing propagation of frailty and stroke risk.

Although much of the focus on associations between frailty and stroke have considered the impact of frailty on stroke, it is important to recognize the impact of stroke on frailty. The neurological deficits following a stroke are likely to exacerbate the phenotypic characteristics of frailty, and prior stroke has been found to be an important factor in the transition from robust to frail, as well as a worsening of a frailty trajectory.^26^ Whether this bi-directional relationship becomes a self-propagating cycle (Figure 3), and whether it may represent a target for intervention, requires further research.

## Impact of frailty on stroke presentation and outcomes

### Stroke presentation, hyperacute therapies, and mortality

Pre-stroke frailty is independently associated with stroke severity in the acute setting, as measured by the National Institute of Health Stroke severity scale (NIHSS).^27^ Mediation analysis in a single center study suggested pre-stroke frailty status is not associated with poorer outcomes directly, but rather the effect is mediated by this association between frailty and stroke severity.^28^ However, other studies report the association between premorbid frailty and early outcomes remains significant after adjustment for stroke severity. In a retrospective single-center study, the CFS was associated with increased 30-day mortality after ischemic stroke after adjustment for age, vascular risk factors, and NIHSS.^15^ Ultimately, large prospective studies are required to elucidate this pathway in terms of the relative contributions from frailty promoting bigger strokes, impaired resilience to withstand the stroke, or a combination of the two.

As well as severity of presentation, frailty may demonstrate a treatment-modifying effect in hyperacute reperfusion therapies, and consequently poorer recovery. In a proof-of-principle study, pre-stroke frailty was independently associated with an attenuated improvement in NIHSS following thrombolysis, with each one-point increase in CFS associated with a reduction of one point in the NIHSS improvement.^15^ Following mechanical thrombectomy, frailty is present in around a third of individuals and is associated with poorer neurological status and increased mortality after 90 days.^29,30^

Both pre-stroke pre-frailty and frailty are independently associated with shorter survival time after stroke in individuals aged under 80 years of age, but not in individuals older than this.^31^ When considering the components of the Fried phenotype, slow walking speed and low grip strength were consistently and independently associated with reduced survival time.^31^

Related syndromes and surrogate markers for frailty may predict outcomes following stroke. Sarcopenia—the loss of skeletal muscle and function that is a major component of frailty—is independently associated with more severe strokes at presentation and poorer outcomes after three months.^32^ Similarly, after controlling for age and stroke type, the only other independent predictor of death after any stroke was poor performance on a timed walk—a surrogate marker of frailty—measured prior to the incident stroke.^33^

### Stroke recovery

Frailty may influence other non-physical aspects of stroke recovery. The premorbid frailty index demonstrates a borderline significant association with the development of post-stroke delirium after adjustment for age, sex, and medication count.^14^ Pre-stroke frailty is independently associated with poorer post-stroke cognition after adjustment for age, delirium, pre-stroke cognitive impairment, and stroke severity.^34^ Pre-stroke frailty phenotypes of slow walking speed and low grip strength are also independently associated with post-stroke cognitive decline and reduced ability to perform activities of daily living.^31^ Such associations have potential repercussions for reduced effectiveness of rehabilitation for individuals with frailty-associated post-stroke cognitive impairment, whilst also representing an important avenue of research to consider whether frailty exerts a treatment-modifying effect on post-stroke cognitive rehabilitation.

Frailty is associated with a marked reduction in self-reported quality of life after stroke, where frail individuals reported poorer quality of life compared to the non-frail group, driven by significant reductions in mobility and self-care categories, after adjustments for age, sex, and NIHSS score.^35^ This study considered the frailty phenotype using self-reported exhaustion, low physical activity, and weight loss from the pre-stroke setting, combined with post-stroke measures of walking speed and grip strength.

Frailty may modulate the response to psychosocial intervention following stroke, with non-frail individuals demonstrating significant improvements in activities of daily living in response to such interventions, whilst no significant improvement (and a trend towards worsening outcomes) was observed in the frail cohort. Similar treatment-modifying effects of frailty upon psychosocial intervention for physical performance and mortality were also observed.^36^

### Discharge destination

In 7258 individuals receiving stroke care in the United States through Medicare, 46.9% of pre-morbidly frail individuals were discharged to a nursing institution, compared to 28% of pre-frail and 18.5% of non-frail individuals. Furthermore, non-frail individuals were 71% more likely than frail (and 16% more likely than pre-frail) to be discharged to in-patient rehabilitation after adjustment for demographics, stroke severity, and co-morbidities.^25^

### Hemorrhagic stroke

In a retrospective single center observational study, frailty was not associated with mortality following spontaneous intracerebral hemorrhage (ICH), nor was frailty associated with post-stroke mRS after adjustment for the Intracerebral Hemorrhage Score.^37^ Higher frailty scores were associated with lower rates of surgical intervention, and in those with more extensive ICH the frailty scores were higher in those who died following withdrawal of care versus those who died despite active management. In those undergoing surgery for spontaneous ICH, frailty was independently associated with higher mortality and poorer longterm neurological recovery 6–8 months after ICH.^38^ In contrast, age was independently associated with poorer neurological recovery but not mortality.

## Frailty and secondary prevention:

Overall the effect of frailty on secondary prevention after stroke has received little attention. However, there has been some consideration of its role in carotid revascularization. In a study of 1,426,343 individuals undergoing carotid revascularization, 59,158 (4.2%) were identified as frail. Compared to non-frail individuals, frailty was independently associated with increased post-procedure mortality, stroke, myocardial infarction, and longer length of hospital stay.^39^ Other studies have reported higher rates of frailty (up to 27.3%), but also supported the independent association of frailty with procedural complications, mortality, and 30-day readmission.^40^ Subgroup analysis suggested that frailty may not be associated with complications and mortality in individuals undergoing carotid stenting, but was unable to determine this definitively.^40^

## Frailty and cerebrovascular pathophysiology

The challenge for frailty research within cerebrovascular disease is to move beyond the reporting of associations to understanding the biological mechanisms through which frailty affects outcomes. Crucially, central and peripheral vascular hemodynamic changes occur in response to ageing, and frailty is associated with impaired cerebral autoregulation.^41^ Distinguishing pathological disease states from “healthy” ageing is paramount for the development of effective interventions. For example, age is negatively correlated with penumbral volume (but not core volume) in individuals undergoing CT perfusion in the hyperacute stroke setting.^42^ However, this work considered only chronological age, not frailty, and it would be advantageous for future work to evaluate the role of frailty in this relationship.

Cross-sectional neuroimaging studies have suggested links between systemic frailty and chronic brain pathophysiology. Frailty is associated with cortical atrophy (predominantly in men),^43^ deep white matter hyperintensities,^19^ severe periventricular white matter hyperintensities, and cortical superficial siderosis.^44^

The presence of white matter hyperintensities (WMH)—but not baseline infarcts or cerebral microbleeds—was associated with frailty progression independently of other small vessel disease markers in a longitudinal population-based study.^45^ In a further longitudinal study, although WMH volume at baseline was associated with a higher likelihood of progression in frailty phenotype severity, no association was found between the progression of WMH volume over the study period and frailty progression, though both the sample size and WMH volume increase over the study period were small.^46^

Features of “brain frailty” (leukoaraiosis, atrophy, and old vascular lesions/infarcts) were associated with poorer functional and cognitive outcomes at 90 days in individuals following ischemic stroke.^47^ Atrophy and leukoaraiosis are also associated with increased 90-day mortality after thrombolysis treatment.^48^ Such imaging criteria may indicate a more “vulnerable” brain with poorer neurological and cognitive reserve, accounting for poorer outcomes, but further work is required to establish the interaction between the frail individual and frail brain, as well as elucidating any underlying biological mechanisms. In addition to the associations of a frail brain with poorer clinical outcome, any attenuation of treatment effect size has also yet to be clearly established.

## Future directions for frailty in stroke medicine

Future work needs to consider the best methods to evaluate frailty in individuals with stroke. The different approaches, relative weightings of different domains of frailty, and scoring systems for evaluating frailty pose challenges for comparing studies and how they may be employed in clinical practice. A single assessment of gait speed and grip strength in the immediate post-stroke setting for a phenotype model may not reflect early neurological recovery or associated complications that may be seen after a stroke, and consequently may over-estimate frailty, and may not be practical in some settings. In clinical practice the quality of pre-morbid data to calculate a frailty index or phenotype is likely to vary, and outside of population research datasets it is unlikely that individuals will have premorbid walking speed and grip strength measured routinely. Frailty indices and the CFS may be more pragmatic and arguably easier to score retrospectively in a general population.

Stroke recovery is multifactorial, consisting of not only physical but also cognitive and psychological recovery. Expansion of the Fried phenotype model to include cognitive frailty, where physical and cognitive deficits frequently co-exist, has been argued to be a better predictor of long-term dependency and death than either domain alone, and may have important implications for identifying those at high risk of post-stroke delirium and mood deficits.^49,50^ Furthermore, an important question relevant for stroke rehabilitation will be whether modifying physical frailty is able to modify cognition, and vice-versa.

Frailty may represent an important target for intervention, either to prevent further deterioration in frailty or possibly to reverse the frailty trajectory in order to reduce its impact on post-stroke outcomes. Multifaceted intervention programmes—including physical, cognitive, and nutritional interventions—have been proposed,^3^ through which interventions hold the most promise in a stroke setting remains unclear.

Frequently, research studies have excluded older people with frailty. Furthermore, there is often a sense of fatalism that results in frailer individuals not being offered the usual evidence-based treatments due to a belief that they will not respond or have a higher risk of adverse events. Consequently, there is a need for robust evidence for prognostication, treatment, and management applicable to frail individuals with stroke who are more representative of the general population seen in clinical practice, and hence necessitate measuring frailty in clinical trials. Incorporation of frailty measures into electronic record systems and national stroke databases may also be advantageous in establishing such trends at a population level.

## Conclusion

Frailty is emerging as an important clinical risk factor for stroke, and is independently associated with a range of poor post-stroke outcomes. Shifting demographics, and the consequent rise in frailty, means that the burden of frailty and its effect on cerebrovascular disease is likely to increase. How best to assess frailty in stroke, attenuate its effects, and incorporate assessment of frailty into treatment decisions, are pressing concerns for both clinical care and research.
